# Effectiveness of photon-initiated photoacoustic streaming in root canal models with different diameters or tapers

**DOI:** 10.1186/s12903-021-01671-4

**Published:** 2021-06-15

**Authors:** Cheng Wen, Yuanyuan Kong, Jian Zhao, Yang Li, Ya Shen, Xuechao Yang, Qianzhou Jiang

**Affiliations:** 1 Department of Endodontics, Affiliated Stomatology Hospital of Guangzhou Medical University, Guangzhou Key Laboratory of Basic and Applied Research of Oral Regenerative Medical, Guangzhou, 510182 Guangdong China; 2 Division of Endodontics, Faculty of Dentistry, The University of British Columbia (UBC), 2199 Wesbrook Mall, Vancouver, V6T 1Z3 Canada

**Keywords:** PIPS, Diameter, Taper, Root canal

## Abstract

**Background:**

This study aimed to compare the use of photon-initiated photoacoustic streaming (PIPS) and conventional needle irrigation (CNI) in conjunction with different concentrations of sodium hypochlorite (NaOCl) to remove *Enterococcus faecalis* (*E. faecalis*) suspended bacteria and biofilms from root canal systems with different diameters or tapers.

**Methods:**

Artificial root canal samples (n = 480) were randomly divided into three groups (n = 160/group). The canals were prepared to fit file sizes #10/.02, #25/.02, or #25/.06. The size #10/.02 group was incubated for seven days. The size #25/.02 or #25/.06 group was incubated for 2 days. A stable biological model of *E. faecalis* infection was established. The root canals were washed with distilled water or with 1%, 2%, or 5.25% NaOCl combined with CNI or PIPS. Bacterial suspensions and biofilms were assessed using an ATP assay kit and fluorescence microscopy. Image-Pro Plus was used to analyse the average fluorescence intensity to determine the most suitable root canal irrigation solution.

**Results:**

In the CNI and PIPS groups, the ATP value of the 5.25% NaOCl subgroup was the lowest, followed by that of the 2% and 1% NaOCl subgroups. The ATP value of the distilled water subgroup was the highest (*P* < 0.05). When the root canal taper was 0.02, the ATP value of the #10/.02 + PIPS group was significantly lower than that of the #25/.02 + CNI group (*P* < 0.05). The average fluorescence intensity of the #10/.02 + PIPS group was lower than that of the #25/.02 + CNI group (*P* < 0.05). When the apical diameter was #25, the ATP value of the 0.02 taper in the PIPS group was lower than that of the 0.06 taper in the CNI group (*P* < 0.05), and the average fluorescence intensity of the 0.02 taper + PIPS group was lower than that of the 0.06 taper + CNI group (*P* < 0.05). PIPS combined with 2% and 5.25% NaOCl effectively improved the long-term antibacterial effect after irrigation and re-culture for 6 h.

**Conclusions:**

Compared with CNI, PIPS has greater ability to remove bacteria in root canals with a small preparation diameter and a small taper. PIPS with 2% and 5.25% NaOCl exhibited superior antibacterial and bacteriostatic effects.

## Background

Eliminating infection within a canal is crucial to the success of root canal treatment, which is currently carried out through a variety of chemomechanical techniques [1]. The purpose of mechanical preparation is to form a good shape in the root canal system, which is conducive to root canal irrigation and filling [2]. In recent years, great progress has been made in the mechanical preparation of root canals; however, these methods are still unable to completely eradicate the bacteria within the root canal [3, 4]. Irrigation is complementary to instrumentation in facilitating the removal of bacteria, debris, and the smear layer [5]. Previous studies have shown that instrumentation and irrigation with NaOCl eliminates bacteria in 50–75% of infected root canals at the end of the first treatment session, whereas the remaining root canals contain recoverable bacteria [6]. Studies have shown that a larger root canal preparation diameter and taper can improve the effectiveness of root canal irrigation, but optimization of the mechanical efficacy of irrigation provided by enlargement of canals may result in weakening of the root structure [7, 8]. With the development of minimally invasive treatments, recent research has focused on methods to reduce mechanical preparation and explore more effective chemical preparation methods [9–11].

Photon-initiated photoacoustic streaming (PIPS) with an Er:YAG laser with low pulse energy (20 mJ) and a short pulse duration (50 μs) has been introduced in root canal treatment to facilitate the removal of bacteria in the root canal system [12]. Akyuz et al. [13] studied the effect of 2.5% NaOCl combined with conventional needle irrigation (CNI), passive ultrasonic irrigation, EndoVac system, Nd:YAG laser, diode laser, Er:YAG laser, and PIPS on *E. faecalis* in the root canal. PIPS combined with 2.5% NaOCl had the best removal effect on *E. faecalis* in the root canal, followed by Er:YAG laser, and the lowest removal efficiency was that of CNI. Akcay et al. [14] studied the penetration depth of PIPS combined with 2.5% NaOCl in dentin tubules and found that compared with CNI, passive ultrasonic irrigation and sonic irrigation, 2.5% NaOCl combined with PIPS infiltrated deeper into the dentin tubules, which showed that PIPS had a strong auxiliary effect in improving the sterilization effect of NaOCl on dentin tubules.

Real teeth have drawbacks, including the need for disinfection/sterilization, ethical and legal challenges in obtaining natural teeth, and anatomical variability [15]. Artificial teeth present no risk of infection, are available in unlimited numbers and are anatomically uniform [16, 17]. Our previous research also showed that PIPS with 2% or 5.25% NaOCl could effectively improve the removal of bacteria in an artificial root canal resin model [18]. However, it is unclear whether PIPS can be used to effectively wash root canals with a small taper and diameter. Therefore, the present study aimed to evaluate the sterilization effect of PIPS laser-activated irrigation with NaOCl in root canals of different diameters and tapers. The null hypotheses tested were as follows: (1) there was no difference in bacterial removal efficacy between PIPS and CNI; (2) there were no differences in bacterial removal efficacy among different concentrations of NaOCl; and (3) increasing the taper and diameter of the root canal had no positive effect on improving the irrigation solution's ability to remove bacteria.

## Methods

### Artificial root canal block

The artificial root canal blocks (n = 480) were made of resin with a curved root canal and a coronal reservoir resembling a pulp chamber (Endo Training Block; Dentsply Maillefer, Ballaigues, Switzerland). The root canals had a 16.5 mm working length with a degree of curvature of 45.2°, a taper of 0.02 and a radius of canal curvature of 6.1 mm [19]. Before the experiment, the model was immersed in 75% alcohol for 30 min, washed with 5 mL of ultrapure water, immersed in 5.25% NaOCl for 30 min, washed with 5 mL of distilled water, and dried at room temperature.

### Canal preparation

The blocks were divided into three groups, and each group was prepared using files with different diameters or tapers (n = 160/group).

#### #10/0.02 group

The root canal was prepared by a K-file (Dentsply Tulsa Dental). It was placed to the working length, and a reciprocal action was used until it fit loosely in the canal.

#### #25/0.02 group

The root canal was prepared by K-file. A #10/0.02 K-file was placed to the working length. A #15/0.02 K-file, #20/0.02 K-file and #25/0.02 K-file were used sequentially until they fit loosely in the canal.

#### #25/0.06 group

The root canal was prepared using MTWO files (VDW, Munich, Germany) adapted to an electric motor (VDW Silver motor; VDW) in rotary movement. The following files were used: size #10/0.04, #15/0.05, #20/0.06 and #25/0.06. The instrumentation was performed in a gentle in-and-out motion, taking the files to the working length.

During instrumentation, the canals were irrigated using 1 mL of distilled water following the use of each instrument. After the instrumentation was completed, the debris in the root canal was subjected to ultrasonic vibration. Then, the root canal opening was dried and sealed with flowing resin (Beautiful Flow Plus F00, Shofu, Japan).

The three groups were subdivided into four subgroups (n = 40) based on the irrigation solution used: subgroup A, distilled water; subgroup B, 1% NaOCl (Sigma-Aldrich Co., USA); subgroup C, 2% NaOCl (Sigma-Aldrich Co.); subgroup D, 5.25% NaOCl (Sigma-Aldrich Co.). Half of the samples in each subgroup **w**ere washed with CNI, and half were washed with PIPS (n = 20).

### Bacterial inoculation of the root canals

*E. faecalis* (ATCC 29212) was grown in brain heart infusion (BHI) broth (Hopebio, Qingdao, China). Single colonies were inoculated in 5 mL of BHI in an aerobic chamber for 24 h at 37 ℃. A 5 × 10^8^ CFU/mL suspension, which was equivalent to 0.5 McFarland, was prepared. Then, 20 μL of the bacterial suspension was added to the root canal systems. The #25/0.02 and #25/0.06 root canal blocks were immersed in 5 mL of fresh BHI for 48 h at 37 ℃ under aerobic conditions. The #10/0.02 root canal blocks were immersed in 5 mL of fresh BHI for 7 days under the same conditions. The bacterial suspension was refreshed every 24 h. Before irrigation, the root canal blocks were washed with 1 mL of distilled water three times. Sterile paper points (Dayading, Tianjing, China) were inserted into the root canal system and left in the canal for 1 min to collect planktonic bacteria. Samples from each canal were collected into separate Eppendorf tubes (S0 sample).

### Root canal irrigation

For CNI, a 30-gauge needle tip (Endo-Eze; Ultradent Products Inc., South Jordan, UT, USA) was inserted into each root canal up to the bend of the root canal system. The root canals were washed for 60 s with 3 mL of irrigation solution.

For PIPS, the tip was left stationary in the pulp chamber and activated while ensuring that the canal and pulp chamber remained passively filled with irrigation solution throughout irrigation. The irrigant was activated by a 2940 nm Er:YAG laser (AT Fidelis; Fotona, Ljubljana, Slovenia) equipped with a handpiece (R14-PIPS, Fotona) holding a 400-μm-diameter quartz tip (XPulse 400/14, Fotona). The tip was applied with 0.3 W, 15 Hz, and 20 mJ per pulse, as recommended by the manufacturer, without water/air spray. The fibre tip was placed in the pulp chamber. The irrigation in the root canals was activated for 30 s with 3 mL of irrigation solution [20].

After irrigation, all samples were washed with 1 mL distilled water to remove residual irrigation solution from the root canal. Subsequently, 10 samples were subjected to similar S0 sampling procedures to obtain S1 samples. The other 10 samples were re-incubated for 6 h. Subsequently, a similar S0 sampling procedure was performed at the root canals to obtain the S2 sample.

### Adenosine 5′-triphosphate (ATP) assay kit analysis

ATP in the root canal system was measured as previously reported [21] using an ATP assay kit (Beyotime, S0026, China) according to the manufacturer's instructions. Each sample of bacteria was collected by sequential placement of sterile paper points in the root canal prepared before irrigation (S0), after irrigation (S1), and after incubation (S2). The paper point was put into the root canal, where it remained for 1 min to obtain a sample of bacteria, transferred to a 1.5 mL Eppendorf tube containing 200 μL of lysis buffer with 0.025 g of glass beads (D3350-01, Omega Biotek Inc, USA), and centrifuged at 12,000 r/min for 5 min at 4 °C for supernatant collection. Then, the ATP detection solution was prepared. Twenty microlitres of ATP detection solution was mixed with 80 μL of diluted solution in a detection tube and placed at room temperature for 5 min. Finally, 20 μL of sample was added to the ATP detection solution and quantified using an ATP fluorescence detector (Lux-T020, China).

### Fluorescence microscope

The processed samples were stained with the LIVE/DEAD BacLight Bacterial Viability Kit (L7012, Life, USA) for 15 min, following the manufacturer’s instructions. Before dyeing, the models were stained with 20 μL of the staining solution in a dark chamber. All the blocks were rinsed with distilled water for 1 min and observed with a fluorescence microscope (BX43, Olympus, Japan). The fluorescence microscopy images were analysed by Image-Pro Plus (version 6.0; Media Cybernetics, Inc., Rockville, MD), which calculated the average fluorescence density to quantify the number of live bacteria. Apical, middle, and coronal thirds were established by marking the roots into 3 levels at 0–3, 3–6, and 6–9 mm from the apical foramen.

### Statistical analysis

Statistical analysis was performed using SPSS Statistics Version 17.0 (IBM SPSS Inc., Chicago, IL, USA). The Shapiro–Wilk test was used to determine whether the test data conformed to a normal distribution, and the Levene test was used to test whether the test data conformed to the homogeneity of variance. When the data conformed to a normal distribution and homogeneity of variance, the results were presented as the means and standard deviations (SDs), and one-way analysis of variance (ANOVA) was used. Otherwise, among-group comparisons were assessed by using the Kruskal–Wallis test; Dunn’s multiple comparison test was used for within-group comparisons. For all tests, statistical significance was set at α = 0.05.

## Results

### Before irrigation

Before root canal irrigation, the ATP value and average fluorescence density were measured. They were not statistically significant in any group (*P* > 0.05).

### After irrigation

#### Root canal models with different diameters

**Fig. 1:**
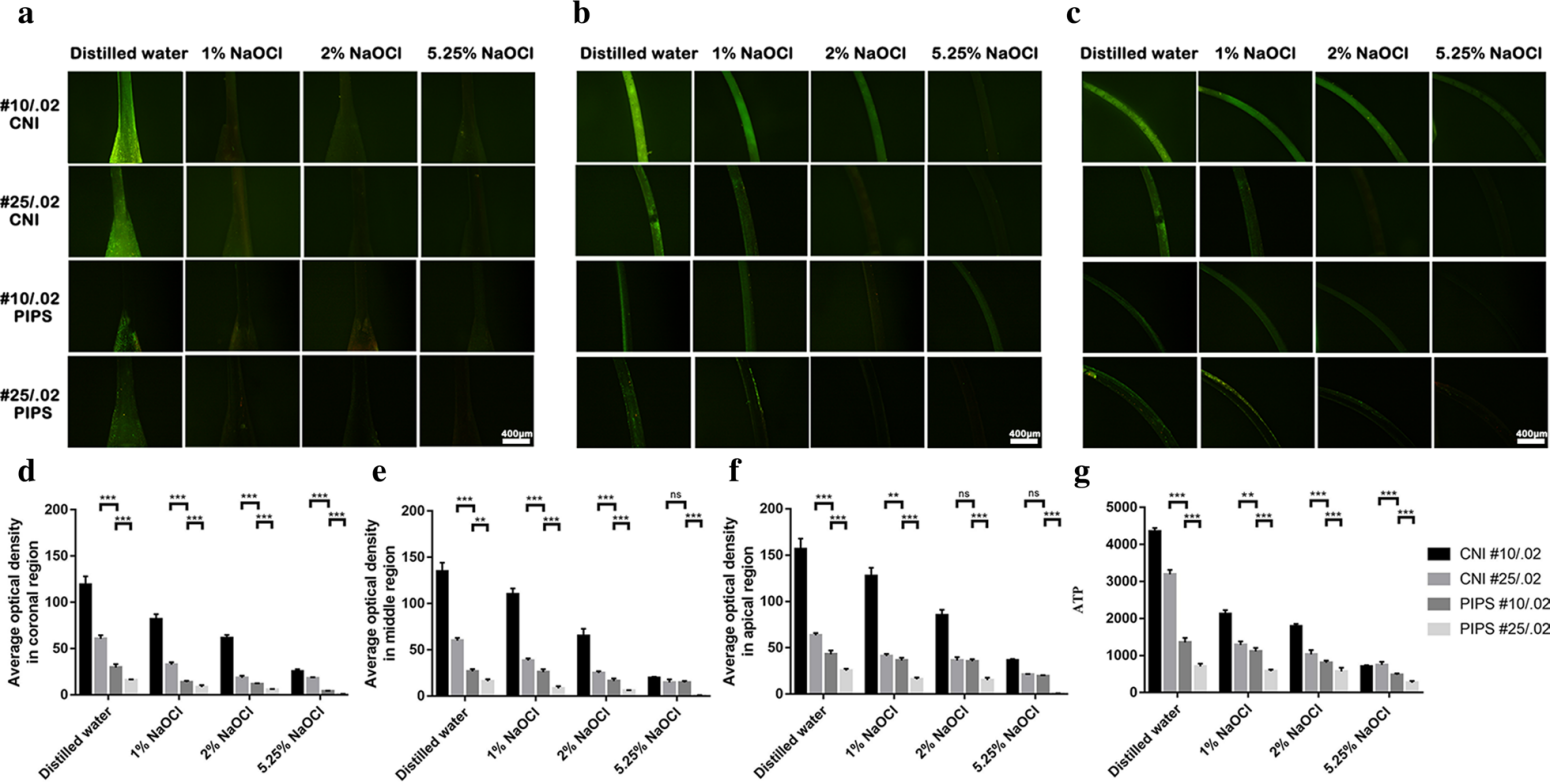
Representative microscopic images of each subgroup prepared by #10/.02 and #25/.02 after irrigation with CNI and PIPS in the coronal region (**a**), middle region (**b**) and apical region (**c**). The value of average fluorescence density in the coronal (**d**), middle (**e**) and apical (**f**) regions. ATP levels are shown in **g**. *p < 0.05, **p < 0.01, ***p < 0.001. Scar bar = 400 μm

ATP levels are shown in Fig. 1g. The canals instrumented to size #10/0.02 in the PIPS group had a significant reduction in ATP value compared with those instrumented to #25/0.02 in the CNI group (*P* < 0.05). The group washed with PIPS exhibited a greater ATP value reduction than the CNI group (*P* < 0.05) with the same diameter.

A larger reduction in average fluorescence density after irrigation was observed in the coronal (Fig. 1a), middle (Fig. 1b and apical regions (Fig. 1c) of the PIPS group than in the CNI group (*P* < 0.05) with the same diameter. The values of average fluorescence density in the coronal (Fig. 1d), middle (Fig. 1e) and apical (Fig. 1f) regions are shown. The canals with size #25/0.02 in the PIPS group had the greatest reduction in bacteria compared with the other groups (*P* < 0.05). The canals instrumented to size #10/0.02 in the PIPS group had a significant reduction in bacteria compared to those instrumented to #25/0.02 in the CNI group (*P* < 0.05), except for the 2% NaOCl subgroup in the apical region and the 5.25% NaOCl subgroup in the middle and apical regions (*P* > 0.05).

#### Root canal models with different tapers

**Fig. 2:**
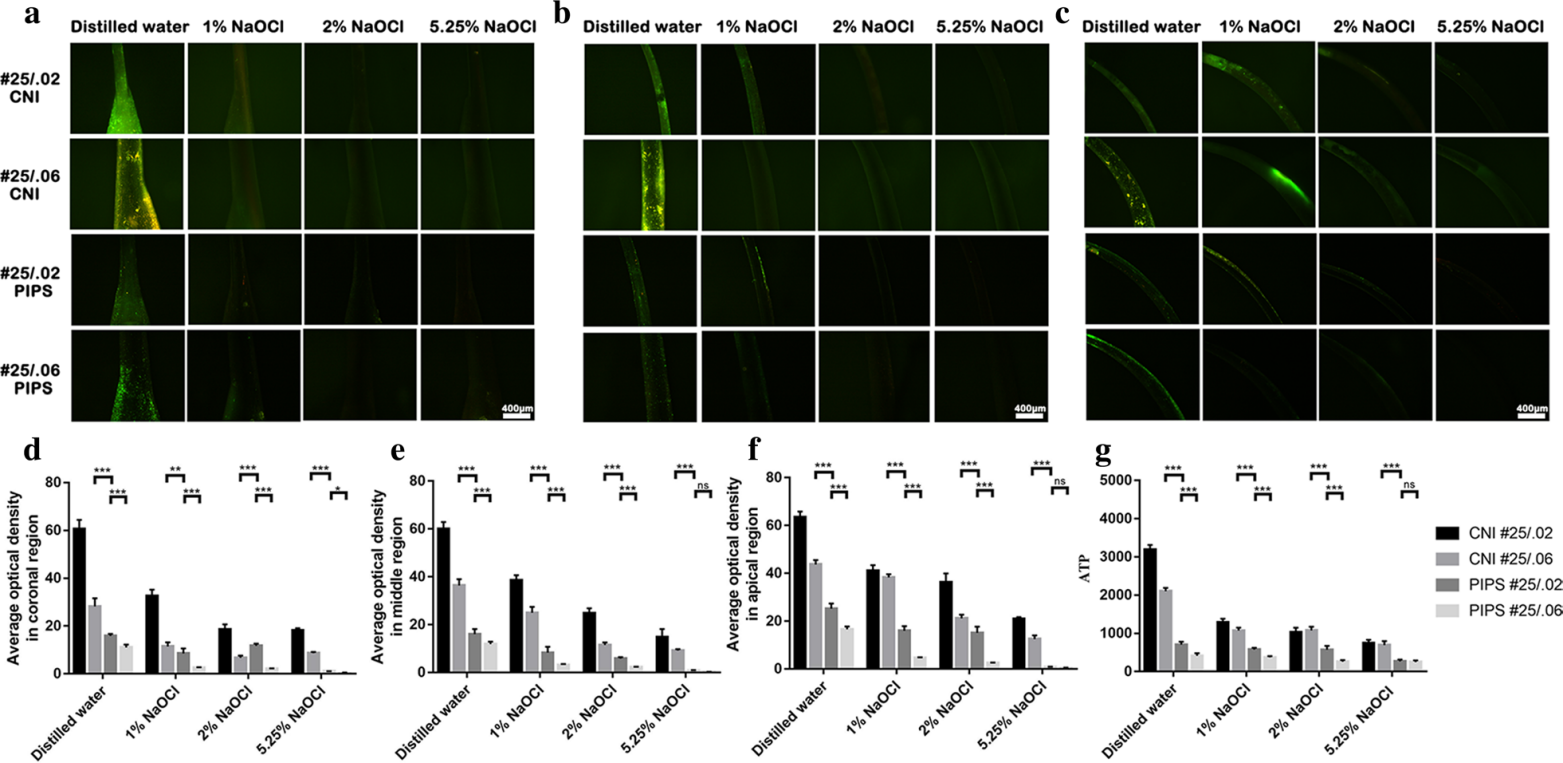
Representative microscopic images of each subgroup prepared by #25/.02 and #25/.06 after irrigation with CNI and PIPS in the coronal region (**a**), middle region (**b**) and apical region (**c**). The value of average fluorescence density in the coronal (**d**), middle (**e**) and apical (**f**) regions. ATP levels are shown in **g**. *p < 0.05, **p < 0.01, ***p < 0.001. Scar bar = 400 μm

ATP levels are shown in Fig. 2g. The canals instrumented at #25/0.02 in the PIPS group had a significant reduction in ATP value compared with those instrumented at #25/0.06 in the CNI group (*P* < 0.05). Irrigation by PIPS resulted in a greater ATP value reduction than irrigation by CNI (*P* < 0.05) in the same taper.

A larger reduction in average fluorescence density after irrigation by PIPS was observed in coronal (Fig. 2a), middle (Fig. 2b) and apical regions (Fig. 2c) (*P* < 0.05) in the same taper. The values of average fluorescence density in the coronal (Fig. 2d), middle (Fig. 2e) and apical (Fig. 2f) regions are shown. The canals instrumented at #25/0.02 in the PIPS group had a significant reduction in bacteria compared to those instrumented at #25/0.06 in the CNI group (*P* < 0.05). The canals with size #25/0.06 in the PIPS group had the greatest reduction in bacteria compared with the other groups (*P* < 0.05).

### After incubation

#### Root canal models with different diameters

**Fig. 3:**
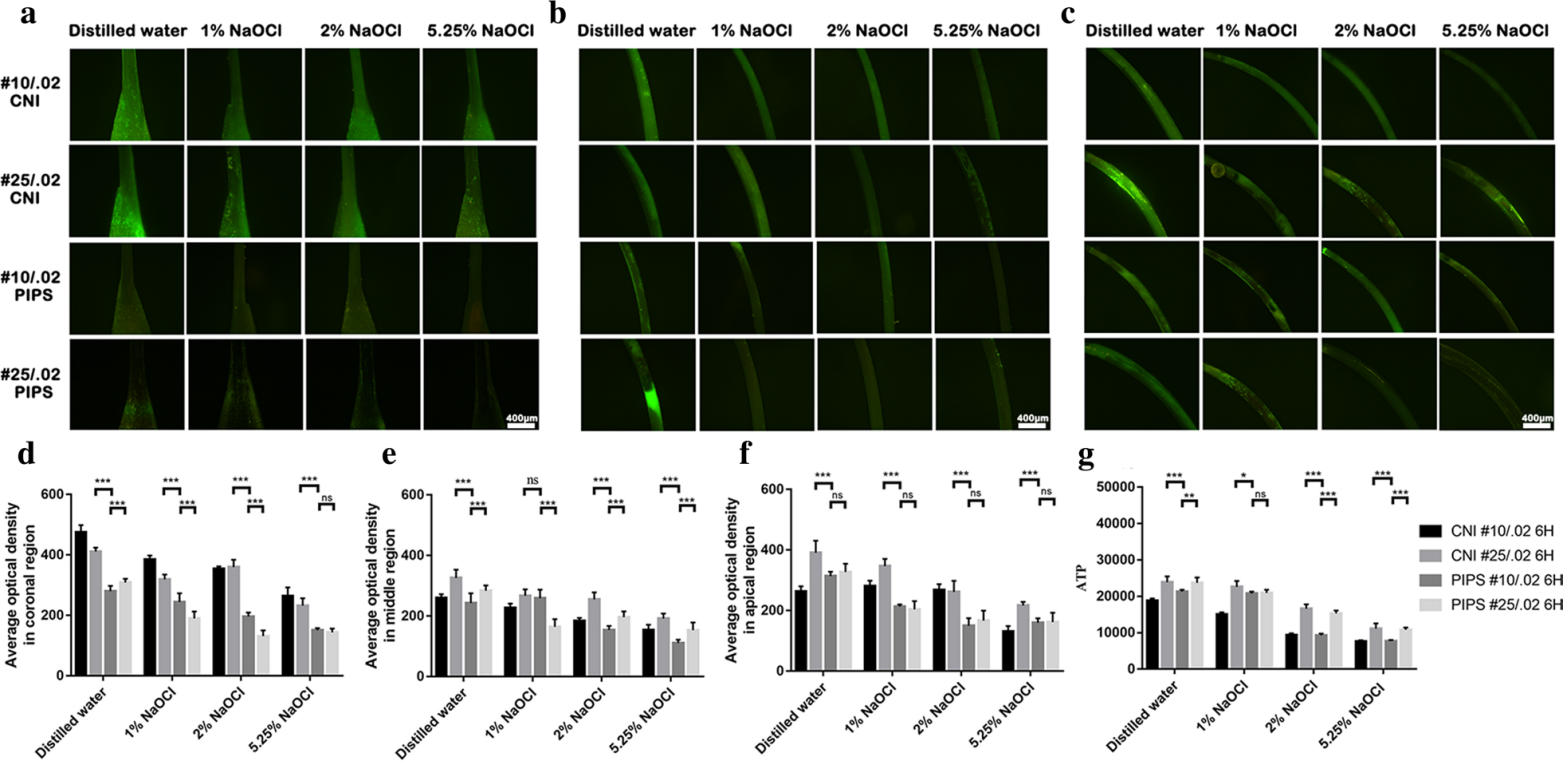
Representative microscopic images of each subgroup prepared by #10/.02 and #25/.02 after incubation for 6 h with CNI and PIPS in the coronal region (**a**), middle region (**b**) and apical region (**c**). The value of average fluorescence density in the coronal (**d**), middle (**e**) and apical (**f**) regions. ATP levels are shown in **g**. *p < 0.05, **p < 0.01, ***p < 0.001. Scar bar = 400 μm

ATP levels are shown in Fig. 3g. After incubation, the ATP value of all subgroups increased significantly. The canals with size #10/0.02 had a lower increase in ATP value than those with size #25/0.02 (*P* < 0.05), except for the 1% NaOCl subgroup with PIPS (*P* > 0.05). The group washed with 5.25% NaOCl with PIPS had the lowest increase compared with the other subgroups (*P* < 0.05).

A greater increase in average fluorescence density after incubation for 6 h was found in the coronal (Fig. 3a), middle (Fig. 3b) and apical regions (Fig. 3c). The values of average fluorescence density in the coronal (Fig. 3d), middle (Fig. 3e) and apical (Fig. 3f) regions are shown. The canals with size #10/0.02 with PIPS had a lower increase in average fluorescence density than those with size #25/0.02 with CNI in the three regions (*P* < 0.05), except for the 1% NaOCl subgroup in the middle region (*P* > 0.05). No significant differences were observed among subgroups in the apical region (*P* > 0.05).

#### Root canal models with different tapers

**Fig. 4:**
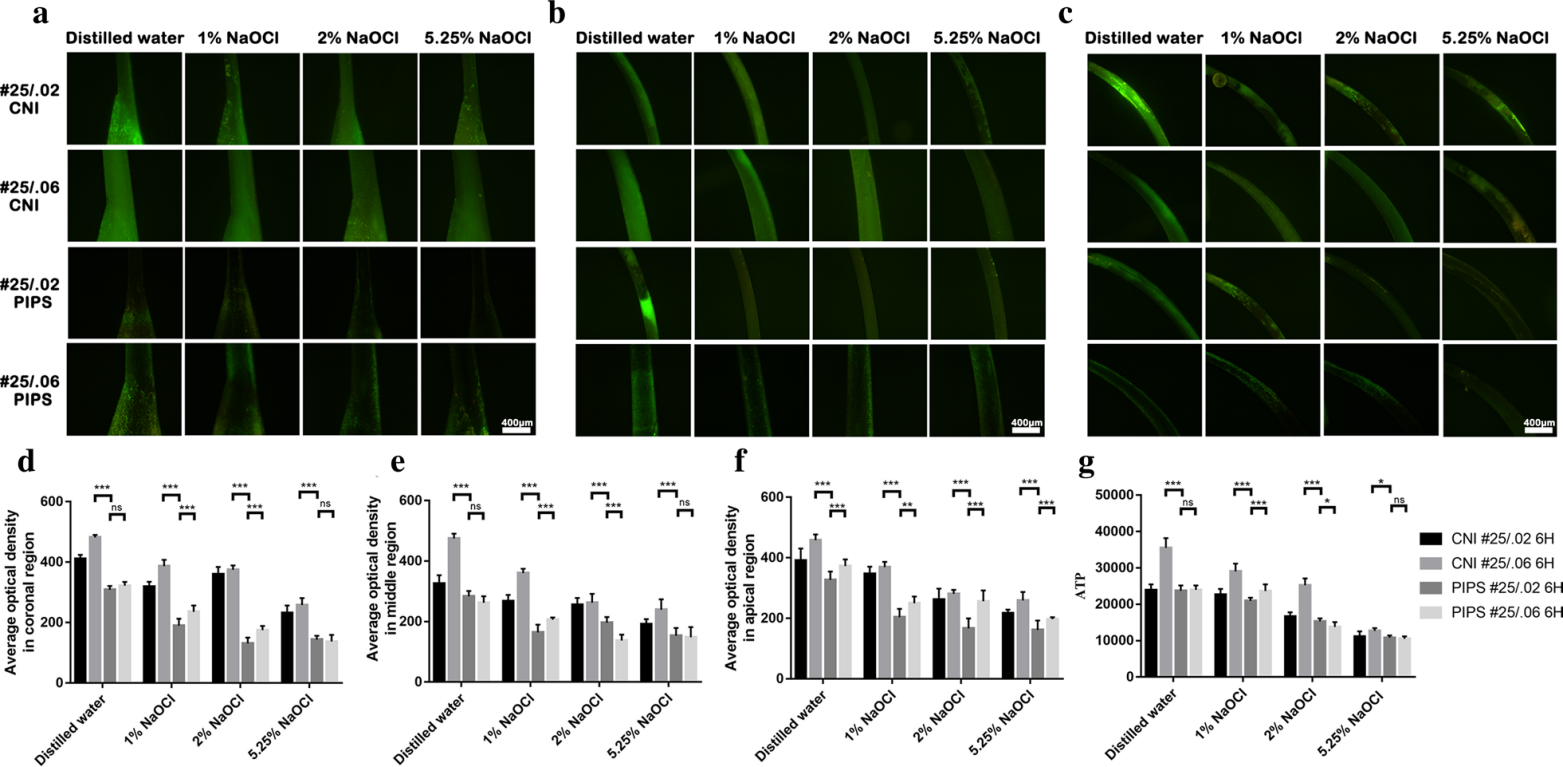
Representative microscopic images of each subgroup prepared by #25/.02 and #25/.06 after incubation for 6 h with CNI and PIPS in the coronal region (**a**), middle region (**b**) and apical region (**c**). The value of average fluorescence density in the coronal (**d**), middle (**e**) and apical (**f**) regions. ATP levels are shown in **g**. *p < 0.05, **p < 0.01, ***p < 0.001. Scar bar = 400 μm

ATP levels are shown in Fig. 4g. The canals with size #25/0.02 had a lower increase in ATP value than those with size #25/0.06 (*P* < 0.05), except the distilled water and 5.25% NaOCl subgroups with PIPS (*P* > 0.05). The 5.25% NaOCl subgroup had the lowest increase in ATP value compared with the other subgroups (*P* < 0.05).

A greater increase in average fluorescence density after incubation for 6 h was found in the coronal (Fig. 4a), middle (Fig. 4b) and apical regions (Fig. 4c). The values of average fluorescence density in the coronal (Fig. 4d), middle (Fig. 4e) and apical (Fig. 4f) regions are shown. The canals instrumented to size #25/0.02 in the PIPS group had a smaller increase than those instrumented to #25/0.06 in the CNI group in the three regions (*P* < 0.05). In the apical region, canals with size #25/0.06 in PIPS had a greater increase than those with size #25/0.02 in PIPS (*P* < 0.05).

## Discussion

*E. faecalis* was chosen because this bacterium is commonly isolated from teeth with failing root canals and is also easy to grow in vitro [22]. Large-taper instruments have been widely used by dentists in recent years; these instruments can prepare the root canal faster and better and make root canal filling more convenient. However, some studies have shown that large-taper root canal preparation instruments increase the diameter of the upper 1/3 of the root canal cavity and make the dentin wall thinner [23]. This can lead to vertical fracture [24], which eventually leads to tooth extraction. The taper of the root canal preparation directly affects the flexural strength of the tooth. If the taper of the root canal preparation is greater than 0.08, the pressure resistance of the root canal wall is reduced. Scanning electron microscopy observations showed that the isolated tooth root with a taper of 0.08 had more surface cracks than the isolated tooth root with a taper of 0.02, 0.04 or 0.06 [25]. The penetration depth of *E. faecalis* in the dentin tubules was approximately 300 μm. If only the root canal preparation was used to remove the biofilm on the wall of the root canal, a large amount of tooth tissue would be lost, which would increase the risk of root fracture. Therefore, it is very important to improve the efficiency of chemical irrigation in the process of root canal therapy. So, in the process of mechanochemical irrigation, can auxiliary irrigation increase irrigation efficiency, reduce excessive root canal preparation and reduce the risk of root fracture?

PIPS is an auxiliary root canal irrigation method that was developed in recent decades. Root canal disinfection by PIPS occurs via the cavitation effect [11], acoustic current effect [26] and thermal effect [27]. When the PIPS laser tip is placed in the medullary cavity, the liquid in the root canal produces large and round water vapor bubbles under the action of the laser, and the total volume of the liquid can be expanded to 1600 times the original volume, which makes it easier for the irrigation fluid to reach the apical region [28]. After the cavitation expands, it becomes unstable and bursts. Shear force is generated on the root canal wall, which reshapes the root canal surface and removes microorganisms in the root canal [29]. Previous studies showed that PIPS was more effective in removing bacteria from the root canal system than conventional needle irrigation [30, 31]. Our previous experimental results also showed that PIPS had greater antibacterial and bacteriostatic effects on *E. faecalis* than CNI in a straight root canal system [18]. However, the bactericidal effect of PIPS on small-taper and small-diameter root canals has not been studied.

When the taper of the root canal was the same, the canals with size #10/0.02 in the PIPS group had a significant reduction in ATP value compared with those with size #25/0.02 in the CNI group (*P* < 0.05). The results showed that PIPS was better than CNI for root canal systems with a smaller taper. Even if the root canal was only dredged with #10/0.02 and washed with NaOCl + PIPS, the sterilization effect was better than that of size #25/0.02 with NaOCl + CNI. After incubation for 6 h, the canals with size #10/0.02 had a lower increase in ATP value than those with size #25/0.02 with PIPS or CNI (*P* < 0.05). The canals with size #10/0.02 in the PIPS group had a significant reduction in bacteria compared with those with size #25/0.02 in the CNI group in the coronal region and middle regions (*P* < 0.05), which was consistent with the ATP experiment. Cheng et al. [1] found that the disinfection efficacy of #15/0.04 washed by the NaOCl + Er:YAG group was similar to that of #40/0.04 washed by the NaOCl group, and the SEM results showed that there was still *E. faecalis* in the 200–300 μm layer, which indicated that in the root canal model (#15/0.04), the Er:YAG laser and NaOCl treatment could effectively kill *E. faecalis* in the root canal, but it could not completely remove *E. faecalis* in deep dentin.

When the diameter of the root canal was the same, the canals with size #25/0.02 in the PIPS group had a significant reduction of ATP compared with those with size #25/0.06 in the CNI group (*P* < 0.05), and canals with size #25/0.02 had a lower increase than those with size #25/0.06 with PIPS or CNI (*P* < 0.05) after incubation for 6 h. When we used PIPS for auxiliary irrigation, even if the root canal was only prepared to #25/0.02, the sterilization effect was significantly better than that in root canals prepared to #25/0.06 without PIPS, which was also consistent with the ATP experiment. Therefore, PIPS could also remove bacteria from root canals with smaller diameters.

These results indicated that PIPS allowed less of the apical preparation to be effectively disinfected, which may prevent the excess loss of dental tissues and conserve the structural integrity of endodontically treated teeth; therefore, this may be a promising procedure for minimally invasive endodontics [11, 28, 32, 33]. The first and third null hypotheses were rejected.

After incubation for 6 h, a lower amount of bacterial growth was observed in the PIPS group, which may be due to the death of a large number of bacteria after irrigation with PIPS. PIPS with 2% and 5.25% NaOCl showed a lower amount of bacterial growth after incubation than the other subgroups. The results of this study were in agreement with the findings of a previous study examining the efficacy of the PIPS method in the destruction of bacterial biofilms [18]. The second null hypothesis was rejected. We observed that more bacteria were produced in the #25/0.06 group. The larger the volume of the culture medium was, the greater the bacterial growth. This showed that the biofilm formed by *E. faecalis* remained in the root canal system even under the mechanical and chemical effects of root canal preparation and irrigation [34]. PIPS combined with NaOCl could not completely remove bacteria in the root canal, which was consistent with some research results [35, 36].

The main advantage of the in vitro root canal model developed was the high degree of standardization of experimental conditions due to exclusion of root canal anatomy and dentine composition as a variable. In this manner, it was possible to execute more runs and to achieve larger test groups. In addition to these advantages, this model also offered the possibility to study other variables, such as canal dimensions, shape and curvature, the effect of grooves, the irrigation time and the use of intracanal medication [31]. The present study has a limitation of design, using an artificial root canal model without the dentinal tubule component of teeth, and may not accurately reflect the strength of adhesion of biofilms to the surface. Additionally, the smooth resin walls of the canals, their perfectly round cross-sectional shape and straight course are different from the clinical situation and might impact fluid dynamics. Therefore, the conclusion of the current study cannot be directly extended to clinical conditions. Further research is needed to complement the results of the present study.

## Conclusions

1. PIPS can improve the ability to remove bacteria in root canals with a small diameter and a small taper.
2. PIPS with 2% and 5.25% NaOCl can have immediate bactericidal effects and long-term antibacterial effects.

## Data Availability

The raw data are available from the authors to any researcher who wishes to collaborate with us. Correspondence should be addressed to Qianzhou Jiang at the following email address. Email address: jqianzhou@126.com.

## Abbreviations

PIPS: Photon-initiated photoacoustic streaming
CNI: Conventional needle irrigation
ATP: Adenosine 5′-triphosphate
BHI: Brain heart infusion
